# Genome-wide identification and functional prediction of novel and drought-responsive lincRNAs in *Populus trichocarpa*

**DOI:** 10.1093/jxb/eru256

**Published:** 2014-06-19

**Authors:** Peng Shuai, Dan Liang, Sha Tang, Zhoujia Zhang, Chu-Yu Ye, Yanyan Su, Xinli Xia, Weilun Yin

**Affiliations:** College of Biological Sciences and Technology, National Engineering Laboratory of Tree Breeding, Beijing Forestry University, mailbox 69, No. 35 Qinghua East Road, Haidian District, Beijing 100083, P.R. China

**Keywords:** Drought, lincRNA, miRNA, non-coding RNA, *Populus trichocarpa*, target mimicry.

## Abstract

Protein-coding genes are considered to be a dominant component of the eukaryotic transcriptome; however, many studies have shown that intergenic, non-coding transcripts also play an important role. Long intergenic non-coding RNAs (lincRNAs) were found to play a vital role in human and *Arabidopsis*. However, lincRNAs and their regulatory roles remain poorly characterized in woody plants, especially *Populus trichocarpa* (*P. trichocarpa*). A large set of *Populus* RNA-Seq data were examined with high sequencing depth under control and drought conditions and a total of 2542 lincRNA candidates were identified. In total, 51 lincRNAs and 20 lincRNAs were identified as putative targets and target mimics of known *Populus* miRNAs, respectively. A total of 504 lincRNAs were found to be drought responsive, eight of which were confirmed by RT-qPCR. These findings provide a comprehensive view of *Populus* lincRNAs, which will enable in-depth functional analysis.

## Summary

A total of 2 542 lincRNAs were identified from *Populus trichocarpa* and some of them play key roles in drought stress tolerance or regulate microRNA through target mimicry patterns.

## Introduction

According to recent studies, more than 90% of eukaryotic genomes are transcribed, yet only 1–2% has protein-coding capacity (Kim and Sung, 2012; Hangauer *et al.*, 2013). To characterize the complete *Populus* genome, all transcripts must be examined. While much is known about *Populus* protein-coding genes, recent studies have suggested that eukaryotic genomes also encode a large number of functional transcripts of non-coding RNAs (ncRNAs), including housekeeping and regulatory RNAs (Chen and Carmichael, 2010; Shuai *et al.*, 2013; Tang *et al.*, 2013). One regulatory ncRNA, long ncRNA (lncRNA), has been reported to be a vital component of eukaryotic gene regulation (Ng and Ng, 2010; Guttman *et al.*, 2011; Nagano and Fraser, 2011; Kim and Sung, 2012; Kornienko *et al.*, 2013).

According to the general location in the genome, there are two types of long ncRNAs; namely, long intron ncRNAs and long intergenic ncRNAs (lincRNAs) (Liu *et al.*, 2012). Bumgarner *et al.* (2009) identified two lincRNAs in yeast that contribute to the control of variegated gene expression. A growing number of lincRNAs are known to be key regulators in higher eukaryotic organisms based on large-scale sequencing. Ulitsky et al. used chromatin marks, poly(A)-site mapping and RNA-Seq data to identify more than 550 distinct lincRNAs in zebrafish (Ulitsky *et al.*, 2011). Large-scale identification of human lincRNAs has been accomplished by analysing various cell types (Khalil *et al.*, 2009; Jia *et al.*, 2010; Cabili *et al.*, 2011; Hangauer *et al.*, 2013; Kumar *et al.*, 2013). A total of 1 119 candidate lincRNAs loci have been identified in the fruit fly, some of which may be important in the nervous system (Young *et al.*, 2012). Li *et al.* (2012) identified 281 novel lincRNAs in chicken skeletal muscle using next-generation sequencing.

lncRNAs play many roles in plants; for example, as precursors of small RNAs (including miRNAs and small interfering RNAs), as a scaffold for multiple protein complexes, and natural antisense transcripts (Xin *et al.*, 2011; Kim and Sung, 2012). Target mimicry is a novel role for plant lncRNAs, such as IPS1 and AT4 of *Arabidopsis* (Franco-Zorrilla *et al.*, 2007; Meng *et al.*, 2012; Todesco *et al.*, 2010; Yan *et al.*, 2012; Axtell, 2013; Wu *et al.*, 2013).

In plants, genome-wide identification of lincRNAs has only been conducted in maize and *Arabidopsis* (Boerner and McGinnis, 2012; Liu *et al.*, 2012). In maize, a computational pipeline using the programming language Python was developed and applied to full-length cDNA sequences to identify, classify, and localize potential lincRNAs. A total of 439 maize lincRNAs were identified (Boerner and McGinnis, 2012). Using a reproducibility-based bioinformatics strategy to analyse 200 *Arabidopsis thaliana* transcriptome data sets, Liu *et al.* (2012) identified 13 230 intergenic transcripts, of which 6480 could be classified as lincRNAs. In *Populus*, studies of regulatory RNAs have focused on miRNAs; however, none have addressed lincRNAs (Li *et al.*, 2011, 2013; Shuai *et al.*, 2013).

In this study, the high-throughput RNA-Seq method, which allows for the sensitive detection of transcripts with low expression and does not depend on current gene annotations, was applied. It is therefore ideal for detecting novel transcripts, especially lincRNAs. Recent studies have shown that lincRNAs are developmentally regulated and responsive to external stimuli (Ben Amor *et al.*, 2009; Xin *et al.*, 2011; Wu *et al.*, 2012). Environmental stressors due to climate change, especially drought stress, could make forests increasingly vulnerable to disease and die-offs (Allen *et al.*, 2010). Drought is known to be an important abiotic stress (Hamanishi and Campbell, 2011). In this study, transcriptome sequencing was conducted in a control library (CL) and drought library (DL) and 2542 lincRNAs were identified. To investigate these novel lincRNAs further, differential expression of lincRNAs between the two treatments was analysed. A total of 504 drought-responsive lincRNAs were identified, some of which were confirmed by RT-qPCR.

In addition, the relationship between lincRNAs and miRNAs was investigated. Some lincRNAs were identified as putative targets and target mimics of miRNAs. Furthermore, the lincRNAs identified here were compared with those identified by previous studies. Very few *Populus* lincRNAs have been identified. Overall, our findings revealed that lincRNAs play key regulatory roles in *Populus*. In this study, a basic annotation set of 2542 lincRNAs is provided, which will increase our understanding of the intergenic functional non-coding genes in plants.

## Materials and methods

### Plant materials and RNA-Seq

Seedlings (female *P. trichocarpa* ‘Nisqually 1’) from tissue culture (5cm tall) were planted in individual pots (15 l) containing loam soil and placed in a greenhouse. After 3 months of growth, they were ~45cm tall. For the drought-stress treatment, different relative soil moisture contents (RSMCs) were used for control and drought conditions. Seedlings from soil with good irrigation (RSMC 70–75%) were used as a control and a low soil water-content level (RSMC 15–20%) was chosen for the drought treatment (Li *et al.*, 2011; Shuai *et al.*, 2013). Leaf water potential (WP) was measured using a PsyPro WP data logger (Wescor). Photosynthetic rate, stomatal conductance, and transpiration rate were measured using the Li-6400 Photosynthesis System (Li-Cor). For another three abiotic stresses, the seedlings grown *in vitro* were untreated or treated with cold (4 °C for 24h), heat (37 °C for 24h), or water (cover the plants with water for 14h). For material harvest, mature leaves from the same position of three individual plants were collected and frozen immediately in liquid nitrogen. For RNA extraction, total RNA was extracted using the standard CTAB method for plants (Chang *et al.*, 1993). Beads with oligo(dT) were used to isolate poly(A) mRNA after total RNA was collected. Fragmentation buffer was added to interrupt mRNA into short fragments. First-strand and second-strand cDNA was synthesized. After that, the short fragments were connected with sequencing adaptors. For PCR amplification, 200–700bp fragments were selected as templates, with respect to the results of agarose gel electrophoresis. Two libraries of CL and DL were submitted to the Beijing Genomics Institute (BGI) for Illumina sequencing (HiSeq™ 2000).

### Assembling RNA transcripts and identifying novel transcriptional units

After filtering out low-quality reads and trimming the adaptor sequences, a total of ~260 million pair-end clean reads were obtained. Clean reads were mapped to the *P. trichocarpa* (version 2.2) genome and gene sequences, respectively, using SOAP2 (Li *et al.*, 2009). Mismatches of no more than five bases were allowed in the alignment. The lincRNA gene structure was optimized according to the read distribution, information of paired-ends, and the genome annotation. The distribution of reads in the genome was obtained by aligning the continuous and overlapping reads to form a Transcription Active Region (TAR). According to paired-end data, the different TARs were connected to construct a potential gene model. The disjunctive parts in the lincRNA gene model were considered lincRNA exons. The other components of the model, which were not detected in sequencing reads, were considered lincRNA introns. Gene models that did not map to the known transcript and were located in the intergenic region (200bp from upstream or downstream genes) were selected for further analysis. To distinguish transcriptional cDNA from genomic DNA contamination, the relatively high expression-level transcripts were subjected to further analysis. Read numbers per base pair of less than 2 were not considered.

After novel intergenic transcriptional units were obtained, they were adapted to four filter processes to identify lincRNA candidates. First, the length of TUs had to be longer than 200bp to exclude small intergenic transcripts. Second, the longest ORF of the TU had to be smaller than 100 AA (the longest ORF predicted by OrfPredictor) (http://proteomics.ysu.edu/tools/OrfPredictor.html) (Min *et al.*, 2005). Both strands of the TUs were used for prediction. Third, to ensure that our results were not influenced by genomic DNA contamination of the cDNA library, the lincRNA candidates must have appeared in both CL and DL. Fourth, these sequences were analysed using BLAST against the miRbase and RepPop to remove miRNA precursors and repetitive elements (Zhou and Xu, 2009; Kozomara and Griffiths-Jones, 2011).

### Validating the non-coding capacity of lincRNAs using CPC and codon usage

The coding potential of all putative lincRNAs were submitted to the CPC (*http://cpc.cbi.pku.edu.cn/*) (Kong *et al.*, 2007). The exonic bases for each transcript in a model were analysed separately and in both orientations (forward and reverse strand). A similar analysis was also performed for 45 033 coding transcripts. A total of 2.3% of genes annotated as protein coding by Phytozome were designated as non-coding by CPC (score less than 0.5) (see Supplementary Table S4 at *JXB* online). If all transcripts within an intergenic model were considered non-coding, it was defined as a lincRNA locus (Young *et al.*, 2012).

Calculation of codon usage was conducted using the longest ORF from OrfPredictor by the online tool (http://www.bioinformatics.org/sms2/codon_usage.html). The Codon Usage database (http://www.kazusa.or.jp/codon/cgi-bin/showcodon.cgi?species=3694) for the *P. trichocarpa* CDS was used as a reference (Nakamura *et al.*, 2000).

### miRNA target and target mimicry prediction

The target was predicted by submitting *P. trichocarpa* miRNAs and the lincRNAs to the psRNATarget (http://plantgrn. noble.org/psRNATarget/) (Dai and Zhao, 2011), with a total of no more than three mismatches and G/U pairs within the lincRNA and miRNA pairing regions. The target mimics were predicted using psRNATarget combined with local scripts and the rules established by Wu *et al.* (2013).

### Differential expression analysis of lincRNAs between the two treatments

The lincRNA sequence reads of the two libraries were normalized to FPKM (fragments per kilobase of transcript per million mapped reads) values in each sample (Trapnell *et al.*, 2010). Calculation of the *P*-value for comparison of lincRNA expression between the two libraries was based on an established method (Audic and Claverie, 1997; Man *et al.*, 2000). The following *P*-value formula was used:

$$\begin{array}{l} p\left(x\text{|}y\right)={\left(\frac{N_{2}}{N_{1}}\right)}^{y}\frac{\left(x+y\right)!}{x!y!{\left(1+\frac{N_{2}}{N_{2}}\right)}^{\left(x+y+1\right)}};\\ p=\text{min}\left\{\sum_{k=0}^{k\leq y}p\left(k\text{|}x\right),\sum_{k=y}^{\infty }p\left(k\text{|}x\right)\right\} \end{array}$$

where *N*_1_ is the total number of reads in the sequencing library of the control, *N*_2_ is the total number of reads in the sequencing library of the drought treatment, *x* is the number of reads for an lincRNA in the control library, and *y* is the number of reads for a lincRNA in the drought treatment library. Specifically, the log_2_ ratio formula was: log_2_ratio=log_2_ (FPKM in DL/FPKM in CL). According to these calculations, lincRNAs with a log_2_ ratio larger than 1 and *P*-value less than 0.001 were considered to be drought responsive.

### Quantitative real-time polymerase chain reaction (RT-qPCR) analysis

In this study, there were 504 drought-responsive lincRNAs. Among them, 60 changed by 4-fold between the control and drought treatments. The 60 lincRNAs were considered the most up- and down-regulated candidates. To validate the lincRNA high-throughput sequencing results, RT-qPCR was performed for eight randomly selected drought-responsive lincRNAs from these 60 lincRNAs. RNA was extracted from leaves using the CTAB method (Chang *et al.*, 1993). All primers used in this study are listed in Supplementary Table S10 at *JXB* online. RT-qPCR was performed using an ABI StepOnePlus instrument. RT-qPCR results were subjected to the following calculations: Sample cycle threshold (Ct) values were determined and standardized relative to the three endogenous control genes (ACTIN, 18S, and HIS), and the 2^–∆∆CT^ method was used to calculate the relative changes in gene expression based on the RT-qPCR data (Livak and Schmittgen, 2001)

## Results

### Physiological characterization of *P. trichocarpa* in response to drought stress

**Fig. 1.**
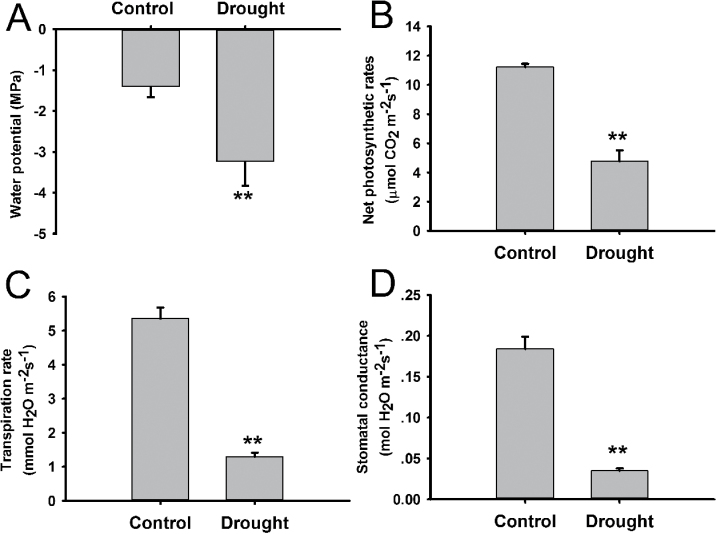
*P. trichocarpa* under control and drought conditions. Leaf WP (A), leaf net photosynthetic rates (B), transpiration rate (C), and leaf stomatal conductance (D) of *P. trichocarpa* under soil water deficiency. Results of the *t* test between control and drought treatments are shown. Values with asterisks (**) were significantly different at the *P* <0.001 level.

*P. trichocarpa* plants were exposed to soil water deficiency at two relative soil moisture contents (RSMC) levels. These levels were set at 70–75% as control and 15–20% for the drought-stress group. The leaf water potential (WP) was detected as a measure of the water status in plants and of the ability of plants to absorb water. A significant decrease (*P* <0.001) in leaf WP from –1.40MPa in controls to –3.23MPa under drought conditions was observed (Fig. 1). At the same time, it was found that the net photosynthetic rates, transpiration rate, and stomatal conductance of leaves under drought conditions significantly decreased compared with the controls (Fig. 1). These physiological differences indicate that plants under drought conditions may show significant changes in gene expression (including lincRNAs). These changes should be identified using RNA-Seq and compared between control and drought conditions.

### Deep sequencing and prediction of novel intergenic transcripts

To identify and investigate the lincRNAs in *Populus* during drought stress, Illumina sequencing of transcripts from CL and DL was performed. A total of ~269 million clean reads (90bp) was obtained (see Supplementary Table S1 at *JXB* online). The average read depth of this sequencing was 57.3-fold that of the whole *Populus* genome. This large data set allowed for the detection of both rare and tissue-specific transcription. Reads of known protein-coding genes accounted for 85% of the total reads. However, the other 15% of reads could not be mapped to known genes and contained regions of high expression within intergenic regions. Transcripts from intergenic regions were then identified. TUs (novel intergenic transcript units) that were 200bp from upstream or downstream genes were explored further. Totals of 11 292 and 11 275 TUs were obtained by high-throughput sequencing from CL and DL, respectively (see Supplementary Table S2 at *JXB* online). These TUs were unknown intergenic transcripts that did not match any known protein-coding transcripts of *P. trichocarpa*.

### Identification of lincRNA candidates

**Fig. 2.**
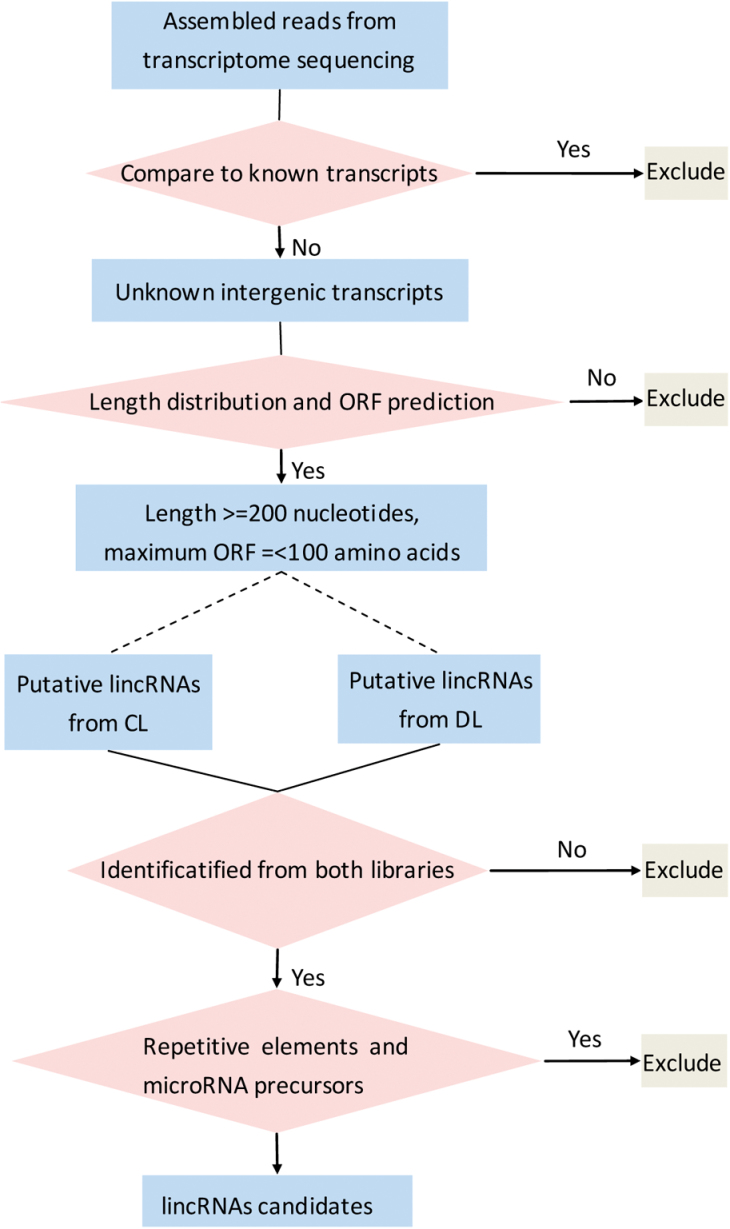
Pipeline from data from RNA-Seq to lincRNA candidates. Sequence reads were assembled and annotated according to the known *P. trichocarpa* transcripts. Unknown intergenic transcripts were filtered using thresholds of ORF length and nucleotide length. After filtering, transcripts found in both libraries (CL and DL) were selected as putative lincRNAs. A total of 2761 putative lincRNA loci were identified from this pipeline.

To identify novel and drought-responsive lincRNAs, these TUs were analysed further using computational and experimental methods. A pipeline for the characterization of *Populus* lincRNAs was constructed (Fig. 2). Totals of 11 292 and 11 275 novel intergenic transcriptional units were analysed in this pipeline. Four filter processes were applied to distinguish lincRNAs from transcript units. To characterize long ncRNAs, the minimum transcript length was 200bp. A total of 220 (CL) and 6 532 (DL) TUs were found to be longer than 200bp in the two libraries. OrfPredictor was used to identify protein-coding regions in TUs (Min *et al.*, 2005), and to calculate the longest possible ORF of each strand. The putative protein-coding RNAs were then filtered using a maximum possible ORF length of 100 amino acids (AA). After these two steps, TUs found in both libraries were selected as putative lincRNAs. After these two steps, 3372 putative lincRNA loci found in both libraries were chosen for the identification and exclusion of repetitive elements and microRNA precursors (see Supplementary Table S3 at *JXB* online). To exclude the repetitive elements, 3372 putative lincRNAs were compared using the RepPop database (Zhou and Xu, 2009). A total of 583 putative lincRNAs showed high similarity with repetitive elements. To identify miRNA precursors from putative lincRNAs, all 352 *Populus* miRNA precursors from miRBase (version 20) were compared with the 3372 putative lincRNAs (Kozomara and Griffiths-Jones, 2011). A total of 28 lincRNAs were identified as miRNA precursors (see Supplementary Table S3 at *JXB* online), which corresponded to ~1% of the total lincRNAs. These selection processes identified 2761 putative lincRNA loci for further investigation (see Supplementary Table S3 at *JXB* online).

### Independent non-coding evidence of the most predicted lincRNAs

As stated above, thousands of putative lincRNAs were distinguished based on a cut-off transcript ORF length. However, it is important to validate the coding status of these lincRNAs loci using an independent method. Therefore, the putative lincRNAs were examined using the Coding Potential Calculator (CPC) filter (Kong *et al.*, 2007). CPC facilitates assessment of the protein-coding potential of large-scale transcripts using sequence features and support vector machines. The protein-coding capacities of TUs on both strands were assessed by CPC. The 45 033 protein-coding transcripts of *P. trichocarpa* (version 2.2) were used as a reference, of which only 2.3% were characterized as non-coding (see Supplementary Table S4 at *JXB* online; Table 1) (Tuskan *et al.*, 2006). The quality of the lincRNA discovery pipeline was demonstrated based on the non-coding potential of 45 033 transcripts. Approximately 92.1% of the 2761 putative lincRNAs were non-coding (CPC score <0.5) (see Supplementary Table S3 at *JXB* online; Table 1). The non-coding rate was higher than those of 6220 and 6532 raw TUs, whose non-coding rates were 90.2% and 90.9%, respectively. A total of 219 putative lincRNA loci classified as coding were discarded. It was also found that the average non-coding potential of lincRNA with more exons was higher than those with fewer exons (Table 1).

**Table 1.** Independent assessment of the non-coding capacity of our lincRNA candidate

Classifications		Number	Non-coding	%
Protein-coding genes in *P. trichocarpa*		45 033	1059	2.3
TU longer than 200bp in CL		6220	5611	90.2
TU longer than 200bp in DL		6532	5938	90.9
lincRNA candidates from pipeline	Total	2761	2542	92.1
	1 Exon	1500	1363	90.9
	2 Exons	765	714	93.3
	>2 Exons	496	465	93.8

Based on this non-coding evidence, the codon usage and GC content of the predicted ORFs in lincRNA candidates was analysed further. This information on coding sequences of *P. trichocarpa* is available in the Codon Usage Database. It was found that the codon usage in the longest predicted ORF in lincRNAs was not correlated with that in the database (see Supplementary Table S5 at *JXB* online). Glutamic acid and aspartic acid content in the CDS was significantly higher than that in lincRNAs. The coding sequences of *Populus* contained 42.6% GC nucleotides, while the mean GC content of predicted ORFs in lincRNAs was 38.8%. The disparity between the protein-coding RNAs and lincRNAs is indicative of different evolutionary pressures in ORFs. These results further support the non-coding potential of these lincRNAs.

### Length and scaffold distribution of lincRNAs

**Fig. 3.**
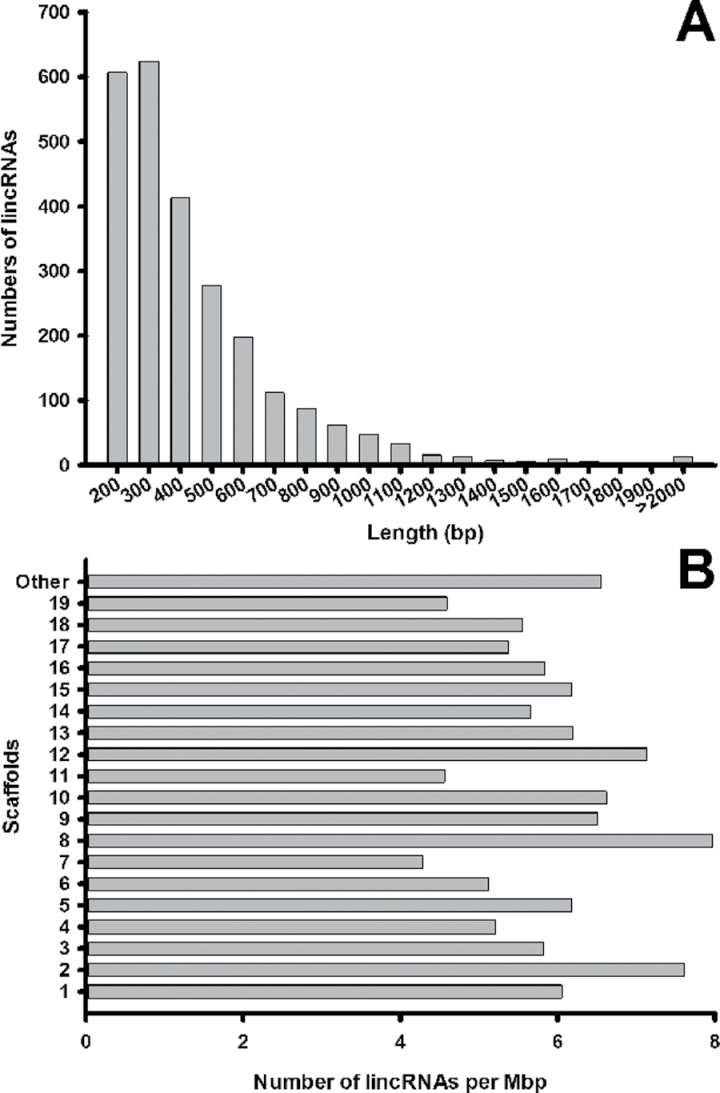
Length and scaffold distribution of lincRNAs. The length distributions of 2542 lincRNAs are shown in (A). The lincRNA number of each Mbp in length on each scaffold in *Populus* is shown in (B). In (B), as the 19 chromosomes of *Populus* from version 2.2 were not well assembled, a scaffold was established called ‘other’ including the remaining unassembled scaffolds.

Based on the above results, 2542 putative lincRNAs were selected for further analysis (see Supplementary Table S3 at *JXB* online). The length distribution of these lincRNA loci ranged from 200bp to 4241bp, yet more than 80% ranged from 200bp to 600bp (Fig. 3A). The most abundant length was 300–400bp. Furthermore, the distribution of lincRNAs in *Populus* scaffolds was examined (as the 19 chromosomes of version 2.2 were not well assembled). In the eighth scaffold, there were 8.3 lincRNAs within every 1 Mbp, which was the highest lincRNA packing density. The seventh scaffold (containing 4.3 lincRNAs per 1 Mbp of nucleotides) had the lowest packing density (Fig. 3B).

### LincRNAs as putative targets of miRNAs

**Fig. 4.**
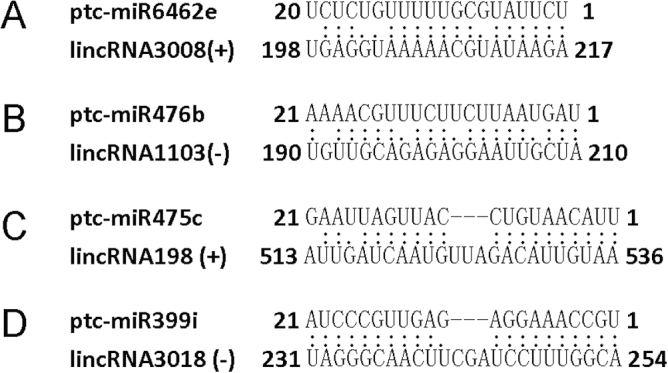
Putative targets and target mimics of lincRNAs. Two lincRNAs as miRNA targets are shown in (A) and (B). Two lincRNAs as target mimics of miRNAs are show in (C) and (D). ‘(+)’, sense strand of lincRNA; ‘(–)’, antisense strand of lincRNA.

The relationship between miRNA and lincRNA is an important issue. Comprehensive miRNA regulation patterns of 90 lincRNAs, some of which are important in breast cancer, were examined in humans (Juan *et al.*, 2013). These miRNAs may play roles in promoting the degeneration of lincRNAs. A total of 30 miRNAs were predicted to target to the sense strand of lincRNAs, while 21 were also found to target the antisense strand (see Supplementary Table S6 at *JXB* online; Fig. 4A, B).

### Functional prediction of lincRNAs as putative target mimics of miRNAs

A mature miRNA leads to the degradation of a specific target; however, this can be inhibited through target mimicry (Hansen *et al.*, 2013). Target mimicry is an important function of lincRNAs in plants. This miRNA–lincRNA relationship has recently been observed in plants (Franco-Zorrilla *et al.*, 2007; Todesco *et al.*, 2010; Meng *et al.*, 2012). *Arabidopsis* IPS1 is the first target mimic identified that is complementary to, and regulates the expression of, miR-399. In this study, the target mimics of these lincRNAs were predicted according to the rules of Wu *et al.* (2013). In total, eight and 12 target mimics were identified on the sense and antisense strand, respectively (see Supplementary Table S7 at *JXB* online; Fig. 4C, D).

**Fig. 5.**
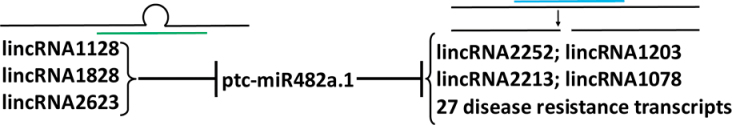
The regulatory mechanism of ptc-miR482a.1 with nine lincRNAs. Ptc-miR482a.1 regulates four lincRNAs (lincRNA1078, lincRNA1203, lincRNA2213, and lincRNA2252) and 27 disease resistance transcripts through degradation. At the same time, three lincRNAs (lincRNA1128, lincRNA1828, and lincRNA2623) regulate ptc-miR482a.1 as target mimics. (This figure is available in colour at *JXB* online.)

Three miRNAs (ptc-miR482a.1, ptc-miR476a, and ptc-miR156c) with different functions (target and target mimicry, respectively) were identified. LincRNA262 and lincRNA2623 is the target and target mimic of ptc-miR156c, respectively. LincRNA1851 has two target mimics (lincRNA20 and lincRNA1795). LincRNA1310 is a target of ptc-miR476a. For ptc-miR482a.1, the relationship was more complex than the other two, and a possible regulatory mechanism is shown in Fig. 5. Ptc-miR482a.1 regulates four lincRNAs (lincRNA1078, lincRNA1203, lincRNA2213, and lincRNA2252) and 27 disease resistance transcripts through degradation. However, three lincRNAs (lincRNA1128, lincRNA1828, and lincRNA2623) regulate ptc-miR482a.1 as target mimics and inhibit its function.

### Identification of drought responsive lincRNAs

**Fig. 6.**
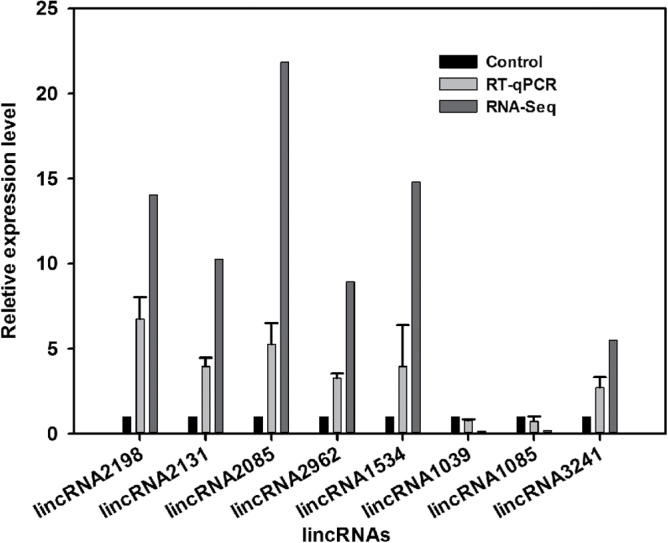
Differential expression analysis of eight lincRNAs under drought stress. RT-qPCR was performed for eight randomly selected drought-responsive lincRNAs from the 60 most up- and down-regulated candidate lincRNAs. Three internal controls (ACTIN, 18S, and HIS) were used for data normalization. The normalized lincRNA levels of the control were set arbitrarily to 1.

To identify drought-responsive lincRNAs from *P. trichocarpa*, the number of normalized lincRNA reads of CL and DL were calculated using FPKM (fragments per kilobase of transcript per million mapped reads) and compared. Based on the sequencing results, lincRNAs with a differential expression of greater than 2-fold and *P*-values less than 0.001 were considered differentially expressed. A total of 504 lincRNAs were identified (see Supplementary Table S9 at *JXB* online), and eight were subjected to experimental validation by quantitative real-time polymerase chain reaction (RT-qPCR). As shown in Fig. 6, the expression patterns indicated by the sequencing and RT-qPCR results of drought-responsive lincRNAs were consistent, although the relative expression levels of all lincRNAs by RNA-Seq were greater than those by RT-qPCR. Therefore, six lincRNAs were identified as up-regulated after drought treatment, while two lincRNAs were down-regulated.

## Discussion

High-throughput RNA-Seq has been used to identify both protein-coding and non-protein-coding transcripts, regardless of whether they are known or unknown (Hangauer *et al.*, 2013). In this study, 2542 lincRNA loci were identified by analysing 269 million sequences. The number of lincRNAs identified by sequencing depends largely on the sequencing depth and species. In the initial screen of more than 20 000 TUs, lincRNAs were selected based on strict criteria. Although this may have excluded many lincRNAs, these 2542 lincRNA loci constitute a reliable set of *Populus* lincRNAs. This is the first study of lincRNA in model woody plants, and will provide a basic overview of lincRNAs in *Populus*.

Due to the inherent limitations of using 90-bp pair-end reads in transcriptome sequencing, the complete structure of a transcript was difficult to obtain. In addition, because our RNA-Seq data lack strand information, it was not possible to determine from which strand the lincRNAs were produced. These limitations are ubiquitous in recent lincRNAs studies using the RNA-Seq method (Li *et al.*, 2012; Kumar *et al.*, 2013). Thus, determination of the full structure and strand information requires improved sequencing technology. Although there are limitations to using lincRNAs, the data can also be valuable. Therefore, lincRNAs from either strand were analysed and their differential expression was evaluated using a fragment of the transcript.

The sequences of 6480 lincRNAs in *Arabidopsis* and 1119 lincRNAs in the fruit fly were compared with 2542 lincRNAs from other species (Liu *et al.*, 2012; Young *et al.*, 2012). Only six lincRNAs identified in this study had homologues in *Arabidopsis*, yet there were no significant matches with the fruit fly (see Supplementary Table S8 at *JXB* online). These results suggest that the majority of lincRNAs identified from our study were not conserved with currently known lincRNAs among plant and animal species. This was also reported in previous studies of other species such as wheat, mouse, and fruit fly (Guttman *et al.*, 2010; Xin *et al.*, 2011; Young *et al.*, 2012).

The FANTOM consortium uses a cut-off of 300 nt (100 AA) to identify putative protein-coding mRNAs (Okazaki *et al.*, 2002). In this study, this ORF threshold was also used to distinguish protein-coding from non-protein-coding transcripts. The two important criteria (ORF and length thresholds) of the pipeline were compared with the number of lincRNAs identified in recent studies (Table 2) (Boerner and McGinnis, 2012; Li *et al.*, 2012; Liu *et al.*, 2012; Young *et al.*, 2012; Hangauer *et al.*, 2013). Four out of six previous studies used an ORF threshold of 100 AA to identify lincRNAs. In chicken, the aggressive criteria of our pipeline resulted in the identification of only a few lincRNAs (Table 2). Based on these results, the criteria (100 AA and 200 nt) used in this work were appropriate. The CPC filter applied here was also used to assess lincRNAs in previous studies (Jia *et al.*, 2010; Boerner and McGinnis, 2012; Young *et al.*, 2012). Based on the low non-coding potential of protein-coding transcripts using CPC in humans and in our study, CPC is a reliable evaluation tool (Jia *et al.*, 2010).

**Table 2.** Comparison of ORF and length thresholds

Species	ORF threshold	Length threshold	lincRNAs found	Reference
Maize	120 AA	200 nt	439	Axtell (2013)
*Arabidopsis*	100 AA	200 nt	6480	Liu *et al.* (2012)
*Populus*	100 AA	200 nt	2542	–
Fruit fly	100 AA	200 nt	1119	Young *et al.* (2012)
Human	100 AA	200 nt	58 465	Hangauer *et al*.(2013)
Chicken	60 AA	300 nt	281	Li *et al.* (2012)

As predicted by Juan *et al.* (2013), lincRNAs are similar in nature to mRNA in that miRNAs can bind lincRNAs and trigger degradation. Previously, lincRNAs were identified as targets of miRNAs in human studies. The repression of lincRNAs could be a novel component of miRNA regulation. In this study, the miRNA–lincRNA interaction were also observed.

Target mimicry is a newly identified miRNA regulation mechanism first studied in *Arabidopsis*. Franco-Zorrilla *et al.* (2007) reported that over-expression of the non-coding gene IPS1 inhibited miR399 and increased expression of the target of miR399. Computational methods have been applied to identify target mimics. However, previous studies focused mainly on target mimics derived from annotated genes; however, no target mimics have yet been identified in *Populus*. In this study, predictions were performed using 2542 lincRNAs and potential target mimics for about 20 miRNAs were identified. Two target mimics (lincRNA432 and lincRNA1174) for miR160 were also identified in *Populus*, which were conserved with the 13 endogenous target mimics identified by Wu *et al.* (2013) in *Arabidopsis* and rice. Ptc-miR482a.1 has been investigated previously and regulates specific disease-resistance proteins in *P. trichocarpa.* This miRNA is known to be responsive to four abiotic stresses (cold, heat, salt, and dehydration) in *Populus* (Lu *et al.*, 2008). Our data suggest involvement of ptc-miR482a.1 in the regulatory network in combination with seven lincRNAs. Three lincRNAs could potentially regulate ptc-miR482a.1 through target mimicry. These lincRNAs maybe associated with abiotic stress tolerance in *Populus*.

**Fig. 7.**
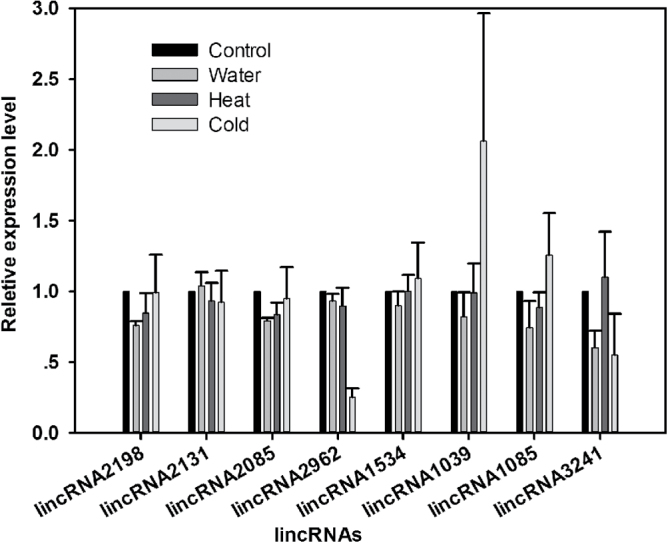
Differential expression analysis of eight drought-responsive lincRNAs under water, heat, and cold stress. Three internal controls (ACTIN, 18S, and HIS) were used for data normalization. The normalized lincRNA levels of the control were set arbitrarily to 1.

LincRNAs are known to respond to biotic and abiotic stresses in plants, such as *Arabidopsis* and wheat (Xin *et al.*, 2011; Liu *et al.*, 2012). Drought-responsive lincRNAs identified in this study were selected using aggressive criteria and were confirmed experimentally. However, some of these drought-responsive lincRNAs were also responsive to other abiotic stresses (water, and cold stresses) (Fig. 7). Drought-induced lincRNA2962 and lincRNA1039 are also down-regulated and up-regulated by cold stress, respectively. LincRNA3241 is down-regulated by water and cold stress.

Interestingly two drought-responsive lincRNAs (lincRNA20 and lincRNA2752) that were target mimics of miRNAs, were identified. Drought-responsive lincRNA20 adsorbed ptc-miR476, which is a specific family in *Populus* according to miRBase (Kozomara and Griffiths-Jones, 2011). However, lincRNA20 is also specific to *Populus*, and may thus represent a *Populus*-specific regulatory mechanism. In addition, drought-responsive lincRNA2752 is a target mimic of ptc-miR169, and could reduce the expression of ptc-miR169. MiR169 is known to regulate the *NF-YA* transcription factor in plants, which is important in drought stress regulation (Ni *et al.*, 2013). This network may be involved in the lincRNA2752-regulation of drought tolerance through miR169 and *NF-YA.* However, the specific regulatory mechanism requires further investigation, and knock out and over-expression of the lincRNA genes in *P. trichocarpa* should be performed to increase our understanding of the regulatory mechanisms.

## Supplementary data

Supplementary data can be found at *JXB* online.

Supplementary Table S1. Summary of *P. trichocarpa* RNA-Seq data.

Supplementary Table S2. 11 292 TUs in the control library and 11 275 TUs in the drought library.

Supplementary Table S3. The 3 372 lincRNA candidates from the pipeline.

Supplementary Table S4. CPC scores of 45 033 *P. trichocarpa* transcripts.

Supplementary Table S5. Comparison of the codon frequency of CDS and lincRNAs in *P. trichocarpa*.

Supplementary Table S6. *Populus* microRNAs target lincRNAs (sense strand and antisense strand).

Supplementary Table S7. *Populus* lincRNAs as target mimics to microRNAs (sense strand and antisense strand).

Supplementary Table S8. Similarities of lincRNAs in *Populus* and *Arabidopsis.*

Supplementary Table S9. Differential expression of lincRNAs.

Supplementary Table S10. Primers designed for RT-qPCR.

## Conflict of interest

The authors have no conflict of interest to declare.

## Supplementary Material

Supplementary Data
