# Supplementary material for: Genome-wide identification and functional prediction of novel and drought-responsive lincRNAs in Populus trichocarpa
Source: J Exp Bot. 2014 Jun 19;65(17):4975–83. doi: 10.1093/jxb/eru256 (PMC4144774; doi:10.1093/jxb/eru256)
Supplement: Supplementary Data [file supp_65_17_4975__index.html]

Genome-wide identification and functional prediction of novel and drought-responsive lincRNAs in Populus trichocarpa — Genome-wide identification and functional prediction of novel and drought-responsive lincRNAs in Populus trichocarpa — Supplementary Data 

# Genome-wide identification and functional prediction of novel and drought-responsive lincRNAs in *Populus trichocarpa*

## Supplementary Data

Data files

**Files in this Data Supplement:**

- Supplementary Data - Supplementary Data
- Supplementary Data - Supplementary Data
