# Supplementary figures and images for: Genome-wide identification and functional prediction of novel and drought-responsive lincRNAs in Populus trichocarpa
Source: J Exp Bot. 2014 Jun 19;65(17):4975–83. doi: 10.1093/jxb/eru256 (PMC4144774; doi:10.1093/jxb/eru256)

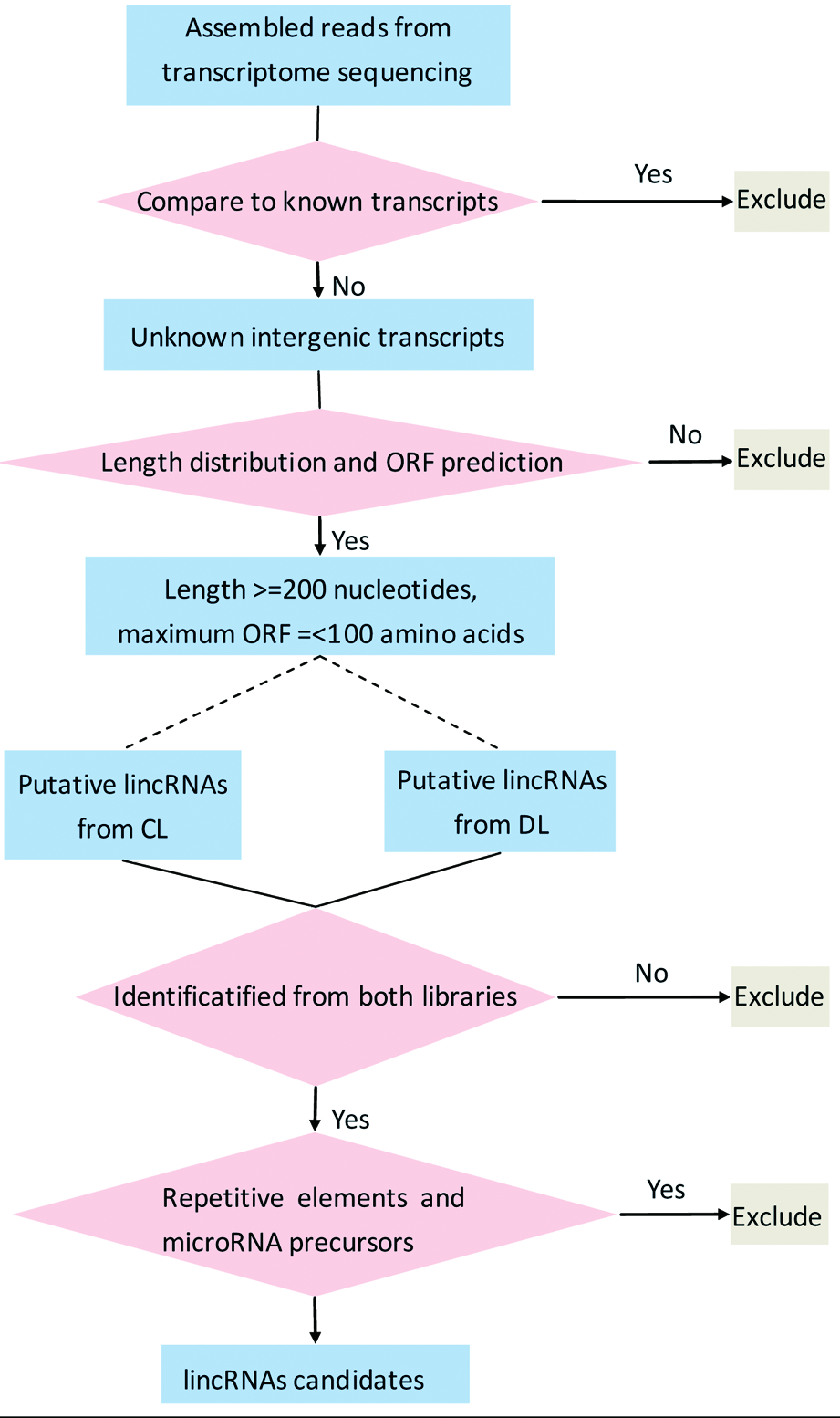

Supplement: Supplementary Data [file supp_eru256_jexbot123174_file002.tif]
